# Antioxidant Vitamins Attenuate Glyphosate-Induced Development of Type-2 Diabetes Through the Activation of Glycogen Synthase Kinase-3 β and Forkhead Box Protein O-1 in the Liver of Adult Male Rats

**DOI:** 10.7759/cureus.51088

**Published:** 2023-12-25

**Authors:** Divaskara Chandran, Selvaraj Jayaraman, Kavitha Sankaran, Vishnu Priya Veeraraghavan, Gayathri R

**Affiliations:** 1 Centre of Molecular Medicine and Diagnostics, Department of Biochemistry, Saveetha Dental College and Hospitals, Saveetha Institute of Medical and Technical Sciences, Saveetha University, Chennai, IND

**Keywords:** foxo, gsk3β, diabetes, glyphosate, antioxidant vitamins

## Abstract

Introduction: Glyphosate is a well-known broad-spectrum desiccant and herbicide. It is an active component used widely in popular weed control products like Roundup (BigHaat Agro Pvt Ltd, Bangalore, Karnataka, India), Rodeo (Corteva, Inc., Indianapolis, Indiana, United States), and PondMaster (PBI-Gordon Corporation, Shawnee, Kansas, United States). However, due to sustained presence, they tend to get deposited in the environmental resources and leach into the living system. It has been shown to develop various cancers and diabetes. However, its impact on GSK-3β (glycogen synthase kinase-3 beta) and FOXO-1 (forkhead box protein O1), both critical proteins involved in the regulation of glucose metabolism and insulin signaling, is unknown.

Objective: The primary objective of this study was to check whether antioxidant vitamins (C and E) can reduce hyperglycemia and hyperinsulinemia in response to glyphosate exposure and the secondary objective was to investigate whether antioxidant vitamins have the capacity to downregulate GSK-3β and FOXO-1-mediated oxidative stress in the liver of glyphosate induced rats.

Methods: We divided the experimental animals into three groups. Group 1 - control rats (animals were injected with olive oil (0.8ml) intraperitoneally), Group 2 - glyphosate-treated rats orally for ten weeks, Group 3 - glyphosate-treated rats received vitamin C and vitamin E. After 30 days of treatment, the animals were anesthetized, sera were separated and used for the biochemical analysis. Liver tissues from control and treated animals were dissected and stored at -20°C for further gene expression analysis. Fasting blood glucose (FBG) was assessed by calorimetric analysis, while serum insulin was measured by enzyme-linked immunosorbent assay (ELISA). Gene expression studies of specific genes (FOXO1 and GSK3) were analyzed by real-time reverse transcriptase-polymerase chain reaction (RT-PCR) analysis.

Results: The expression level of FOX01 and GSK3β genes was higher in glyphosate-induced animals compared with the control group but was reduced significantly (p＜0.05) upon treatment with antioxidant vitamins (C and E). Other biochemical parameters, including FBG, serum insulin, and antioxidant enzyme assays, also showed that antioxidant vitamins reduce glyphosate-induced insulin resistance and type-2 diabetes.

Conclusion: The current study provides in vivo experimental evidence that antioxidant vitamins (C and E) reduce the glyphosate-mediated development of type-2 diabetes risk via the downregulation of FOX01 and GS-3β mRNA expression in the liver. Hence, vitamins C and E may be considered as therapeutics for the treatment of diabetes.

## Introduction

Pesticides are economically significant substances in agricultural products and the environment, causing risks to both human and animal health [1,2]. Glyphosate is one of the most widely used organophosphate herbicides globally. It is active in well-known weed control management agents such as Roundup (BigHaat Agro Pvt Ltd, Bangalore, Karnataka, India), Rodeo (Corteva, Inc., Indianapolis, Indiana, United States), and PondMaster (PBI-Gordon Corporation, Shawnee, Kansas, United States). Numerous farmers rely on its application within the realm of food production [3]. Due to its high solubility and widespread application, glyphosate is regarded as a non-discriminatory, versatile, and effective herbicide. The half-life of glyphosate ranges between 0.8 to 150 hours [3]. From a chemical perspective, glyphosate is a relatively uncomplicated molecule categorized as an organophosphorus compound, specifically, a phosphonic acid. This compound originates from the formal oxidative combination of the methyl group found in methyl phosphonic acid with the amino group of glycine. It is analogous to the natural amino acid glycine, featuring a fundamental amino group and a highly ionized 126-phosphate group. Consequently, glyphosate becomes an exceedingly polar and amphoteric molecule [4]. Presently, this herbicide is extensively utilized by both developed and developing nations. The extensive use of glyphosate may lead to its accumulation due to its long-term life, and it is not easy to purify its effluents from environmental sources. The risk factors and concerns regarding glyphosate usage have led to many controversies, like whether it has to be banned, restricted, or promoted [4,5]. 

Glyphosate has been shown to cause insulin resistance. Its impact on the pancreas may be involved with type-2 diabetes mellitus (T2DM), which leads to the dysfunction of various signaling mechanisms that lead to either overexpression or reduced expression of multiple genes involved in glucose utilization, thus accelerating hyperglycemia [6,7]. Free radicals are the molecules the cells release during oxidative stress by various biological processes and develop multiple diseases, including T2DM [8]. Moreover, these free radicals can be scavenged by either natural antioxidants or synthetic antioxidants. Some natural antioxidants in the human body are antioxidant enzymes (superoxide dismutase, catalase, peroxidase, and reductase) and vitamin C. Oxidative damage is majorly involved in the progression of numerous pathogenesis such as diabetes [9,10]. An enhanced free radical scavenging system in the host may ameliorate oxidative damage and inhibit or halt the prognosis of pathogenesis [11,12]. Carotenoids, vitamin C, and vitamin E are well-known naturally available potential free radical scavengers. Along with Cytochrome p450, two other enzymes, namely, glutathione transferases (GST) and glucose-6-phosphate dehydrogenase (G6PD), also play a crucial role in detoxification mechanisms, which are negatively influenced by Roundup [13]. An in vitro study investigated the adverse effects of glyphosate against various hepatic enzymes. The study results showed alterations in the levels of these enzymes upon glyphosate exposure [14]. 

Glycogen synthase kinase 3 (GSK3) is a serine/threonine protein kinase enzyme that is crucial in adding phosphate molecules to serine and threonine amino acid residues. GSK-3β (glycogen synthase kinase-3 beta) and FOXO-1 (forkhead box protein O1) are both important proteins involved in the regulation of glucose metabolism and insulin signaling, making them relevant to understanding diabetes. Antioxidant vitamins are crucial in safeguarding the body from the adverse effects of various free radicals. This is achieved by enhancing the activity of enzymic antioxidants as coenzymes in their reduced forms or by directly neutralizing free radicals.

It has been shown that antioxidant vitamins (C and E) reduce insulin resistance against an endocrine disruptor, diethylhexyl phthalate (DEHP), in animals. A few studies have shown that glyphosate exposure negatively influences glucose homeostasis [15,16]. However, it is unknown whether glyphosate can induce hyperglycemia by interfering with GSK-3β and FOXO-1, proteins involved in the regulation of glucose metabolism and insulin signaling in hepatocytes. Hence, in the present study, we aimed to analyze the possible role of antioxidant vitamins (C and E) in reducing the glyphosate-mediated development of hyperglycemia and hyperinsulinemia by downregulating the expression of GSK-3β and FOXO-1 mRNA in the liver. 

## Materials and methods

Animals and maintenance

For this ethically authorized research study (Approval No: 086, 2021), male albino Wistar rats aged 100 days and weighing 180-200 g were housed in a controlled setting at the Biomedical Research Unit and Laboratory Animal Centre (BRULAC), Saveetha Dental College (SDC), Saveetha Institute of Medical And Technical Sciences (SIMATS), Tamil Nadu, India. 

Experimental design

Animals were divided into three groups, each group comprising six animals. Group 1 served as control rats (vehicle control, normal control rats were given 0.8 ml of corn oil orally) for the experimentation; Group 2 animals were induced with glyphosate at a dose of 100g/kg body weight; Group 3 glyphosate-induced animals were treated with vitamin C (100mg/kg body weight) and vitamin E (50mg/kg body weight) for 30 days (vitamin E was dissolved in olive oil while vitamin C was dissolved in water). After the treatment, animals were anesthetized with sodium thiopental (40mg/kg body weight, intraperitoneally), and the blood was collected. The sera were separated and stored at −80 °C for experimentation. The liver tissue was dissected out from all three experimental groups and used for assessing various parameters.

Assessment of fasting serum insulin

Fasting serum insulin was assayed using an ultrasensitive rat insulin ELISA kit obtained from Crystal Chem, Inc. (Elk Grove Village, Illinois, United States). The range of detection was 0.1 - 64 ng/ml. The percentage cross-reactivity of insulin antibody to rat insulin was 100%. The intra-assay coefficient of variation was ≤10.0% and the inter-assay coefficient of variation was ≤10.0%. Results were expressed as µIU/ml.

Fasting blood glucose (FBG)

FBG was quantified using On Call Plus blood glucose test strips (ACON Laboratories, Inc., San Diego, California, United States) and expressed as mg/dL. 

mRNA expression analysis of GSK3β and FOXO1 by reverse transcriptase-polymerase chain reaction (RT-PCR)

In order to measure the mRNA levels of insulin metabolic signaling, we employed the RNAiso plus reagent (Takara Bio, Inc., Kusatsu, Shiga, Japan) to extract RNA from the adipose tissue of both control and diabetic rats. The RNA pellet was homogenized, centrifuged, and resuspended in a 70% ethanol solution, and the RNA pellet was dissolved in the diethylpyrocarbonate (DEPC) water and quantified at 260/280 nm. The purity of total RNA was found to be 1.8-2.0. Further, a reverse transcriptase enzyme was utilized to generate complementary DNA (cDNA) by means of the reverse transcription process. Using the SYBR master mix (Thermo Fisher Scientific, Waltham, Massachusetts, United States) and gene-specific primers, quantitative real-time RT-PCR (qRT-PCR) analysis was performed (CFX96 Touch Real-Time PCR detection system, Bio-Rad Laboratories, Hercules, California, United States) to quantify the relative amount of mRNA. Thermal cycling conditions for the qRT-PCR analysis we as follows: initial denaturation at 95 °C for 5 minutes followed by 40 cycles of 95 °C for 30 seconds, 59-60 °C for 30 seconds, and 72 °C for 30 seconds. As invariant control, we used rat β-actin in this study.

Statistical analysis

The results were presented as the mean ± standard error of the mean (SEM) from three distinct experiments conducted in triplicate. Statistical analysis involved the utilization of one-way Analysis of variance (ANOVA), with p-values less than 0.05 being considered indicative of statistically significant findings. For two comparisons, the Student 't" test was performed.

## Results

**Figure 1:**
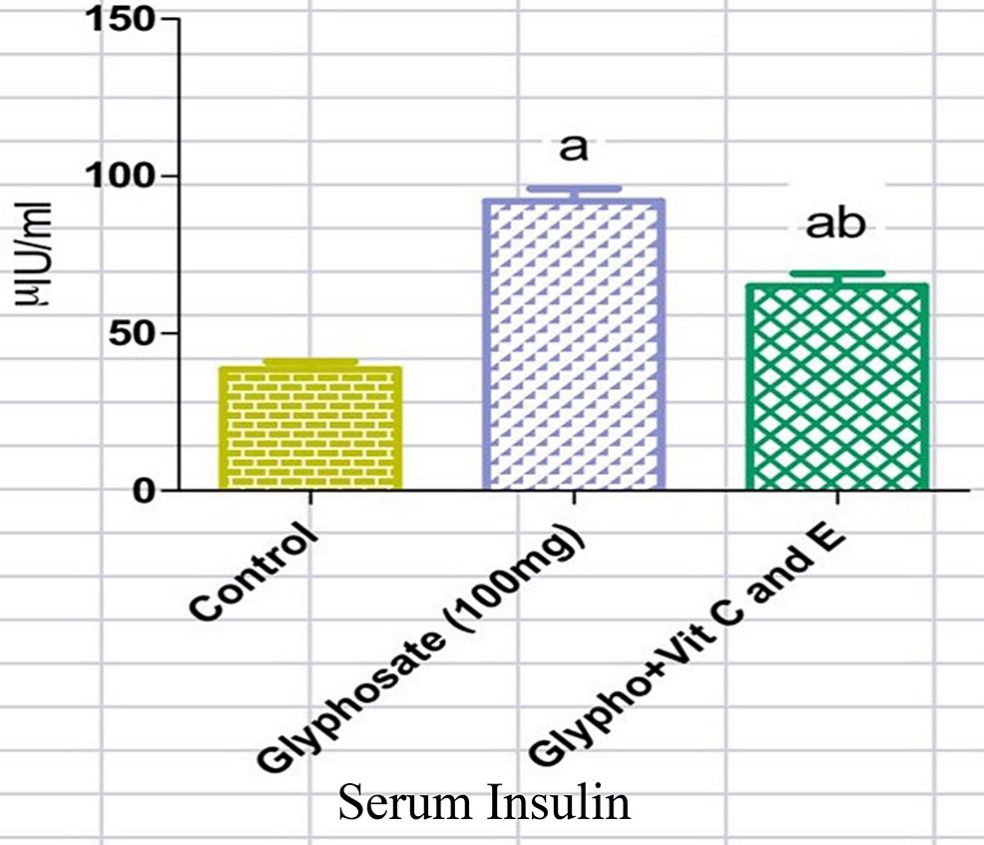
Serum insulin level Changes in the levels of serum insulin in normal, glyphosate, and vitamin C and E treated rats. Values are given as mean ± standard error of the mean. Significance at p<0.05. 'a': significantly different from the control group, 'ab': significantly different from the control and glyphosate-treated groups.

**Figure 2:**
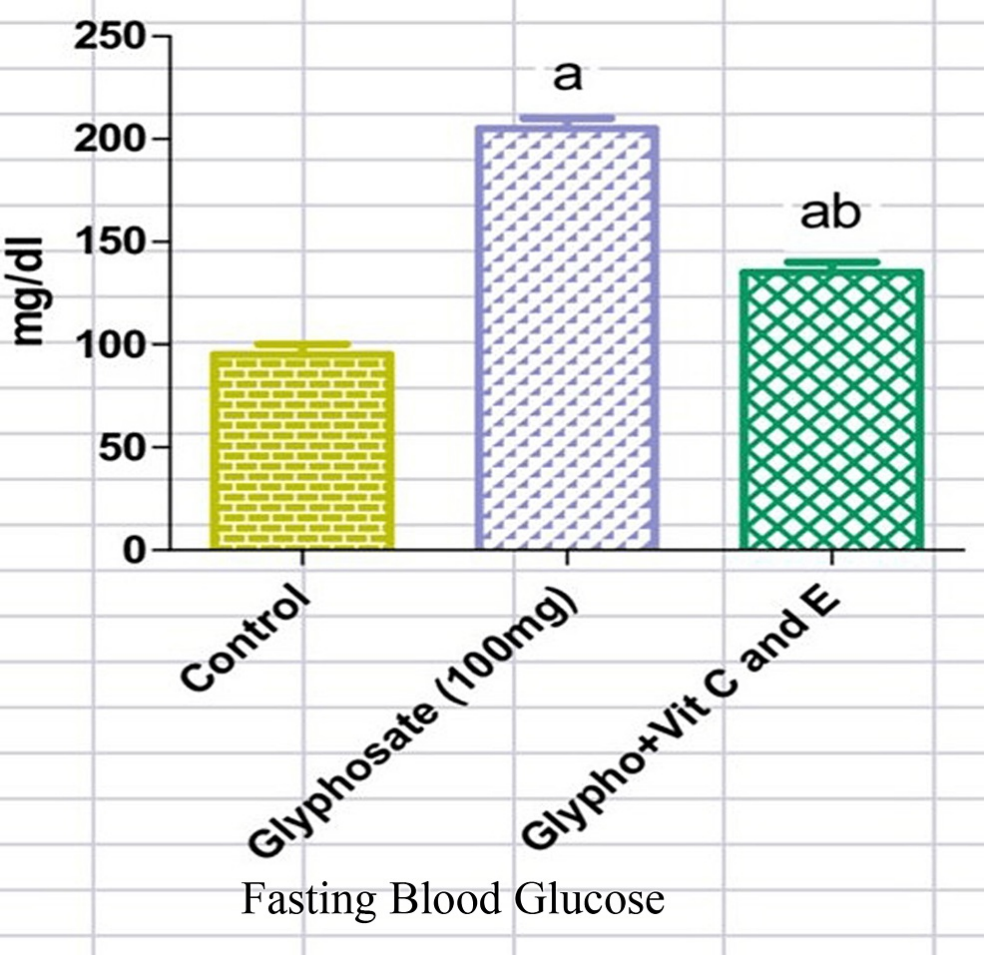
Fasting blood glucose (FBG) level Changes in the FBG in normal, glyphosate, and vitamin C and E treated rats. Values are given as mean ± standard error of the mean. Significance at p<0.05. 'a': significantly different from the control group, 'ab': significantly different from the control and glyphosate-treated groups.

**Figure 3:**
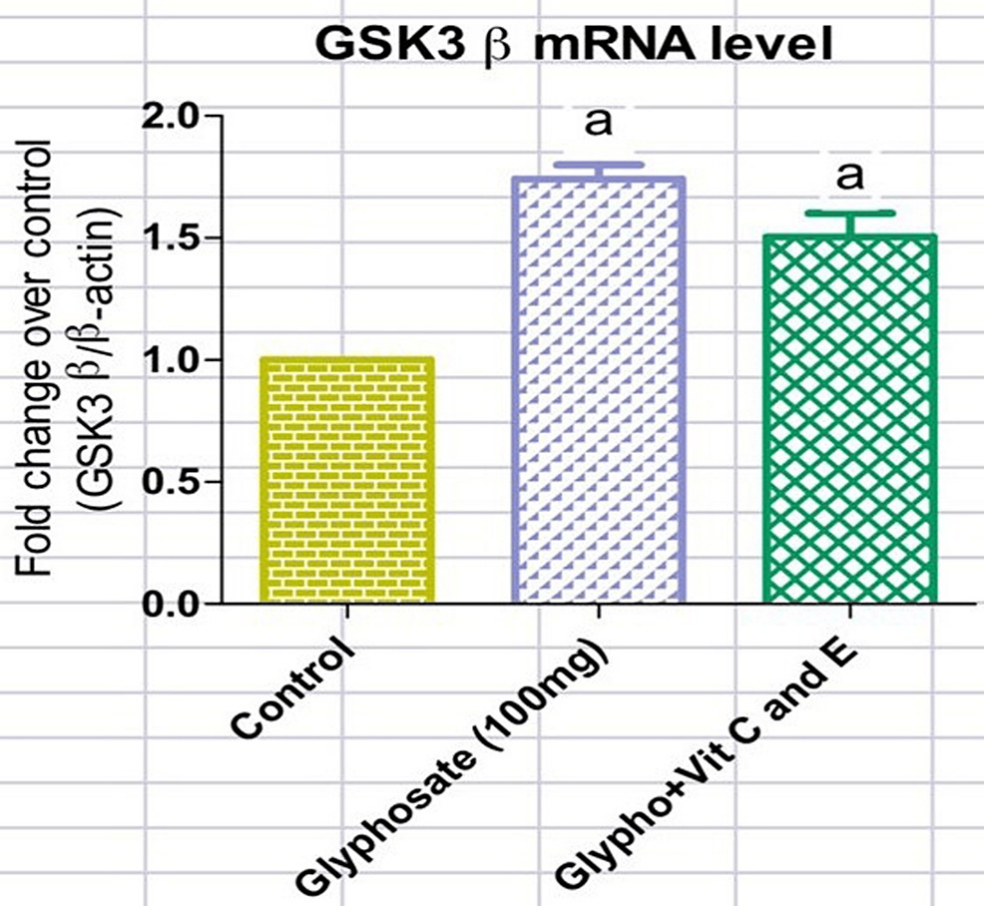
mRNA expression of glycogen synthase kinase-3 beta (GSK-3β) Changes in the mRNA levels of GSK3β in normal, glyphosate, and vitamin C and E treated rats. Values are given as mean ± standard error of the mean. Significance at p<0.05. 'a': significantly different from the control group.

**Figure 4:**
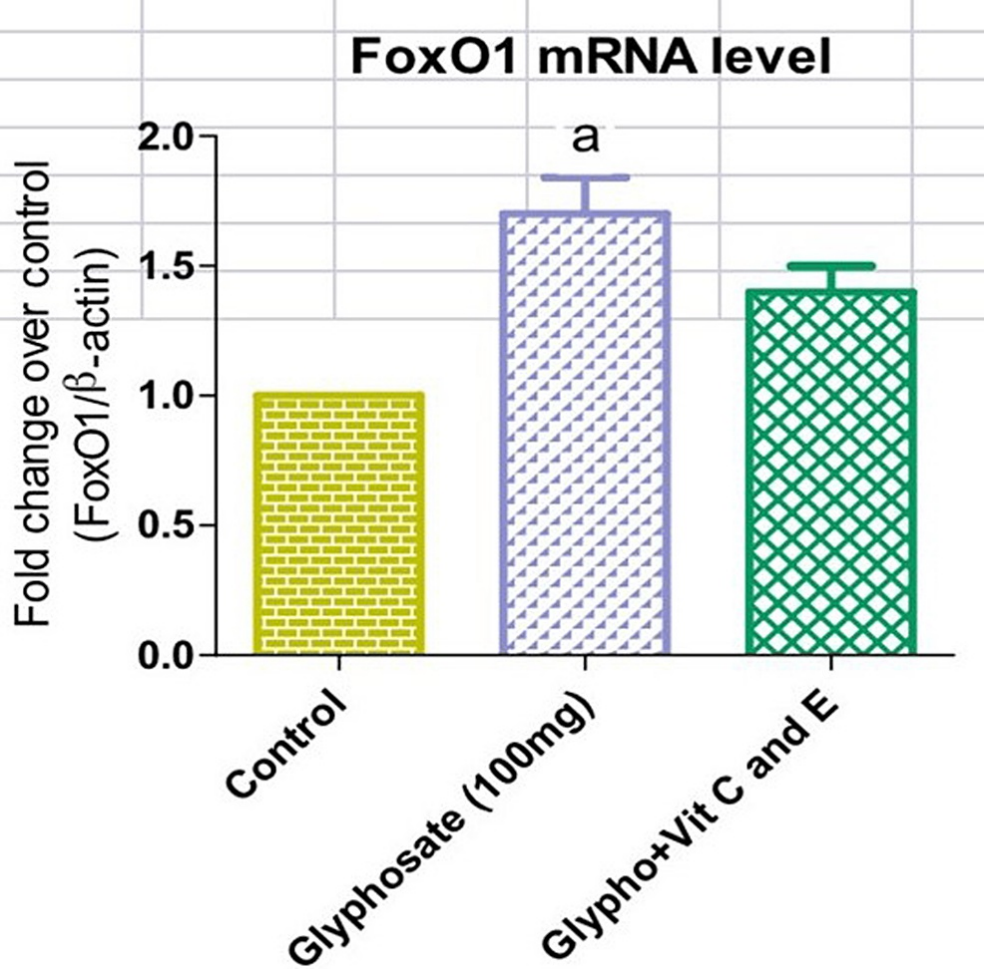
mRNA expression of forkhead box protein O1 (FOXO1) Changes in the mRNA levels of FOXO1 in normal, glyphosate, and vitamin C and E treated rats. Values are given as mean ± standard error of the mean. Significance at p<0.05. 'a': significantly different from the control group.

From Figures 1, 2, it is clear that upon glyphosate exposure, there was an alteration in the blood glucose (218mg/dl) and serum insulin (95micro IU/ml) levels. There was a rise in the serum insulin level in the glyphosate-induced animals, while those treated with antioxidant vitamins showed a significant decrease in the serum insulin (57 micro IU/ml) levels (Figure 1). Likewise, the level of FBG (218mg/dl) was elevated in the glyphosate-exposed groups whereas in the treatment group with vitamins C and E, there was a marked reduction in the circulating FBG (130mg/dl) levels (Figure 2). From Figures 3, 4, it is clear that upon glyphosate exposure, there was marked dysregulation in gene expression of GSK3β (0.75 fold increase) and FOXO1 (0.6 fold). Compared to the control, there was an increase in the mRNA expression of GSK3β in the glyphosate-exposed animals, and in the treatment group, there was reduced mRNA expression (Figure 3). Likewise, there was an upregulation in FOXO1 expression in the glyphosate-induced animals when compared to the control, but upon treatment with antioxidant vitamins, a decrease in the gene expression (0.5 fold decrease) was noted (Figure 4).

## Discussion

Exposure to harmful substances such as pesticides in the environment can lead to metabolic imbalances and oxidative stress in the biological systems. One of these, which is crucial in the emergence of numerous metabolic illnesses, is glyphosate. Besides, in the last 25 years, the global usage of glyphosate has continued to rise by more than tenfold. In terms of its chemical structure, it lacks functional groups capable of establishing stable bonds with DNA. Based on the Deductive Estimation of Risk from Existing Knowledge (DEREK) findings, glyphosate is not considered to pose a risk of chromosomal damage or mutagenicity [17]. FBG is the key parameter for confirmation of the diabetic condition. In the present study, in order to identify whether vitamins C and E can reduce glyphosate-induced hyperglycemia, FBG levels were assessed among the three groups. The results showed that in the glyphosate-induced diabetes conditions, FBG was high, and when vitamins C and E were induced, FBG concentration was near that of the near to control level. These observations clearly show that vitamins C and E have a definite role in reducing hyperglycemia that was induced by glyphosate in rats. Further, we intended to check hyper-insulinemic insulin resistance that was responsible for the rise in FBG levels; we measured serum insulin concentration in response to antioxidant vitamins in glyphosate-exposed rats. Results of these parameters showed that in the glyphosate-induced group, serum insulin level was high when compared to control rats. However, the vitamins C and E administered group showed a significant reduction in the serum insulin level, which suggests that vitamins C and E lower the glyphosate-induced hyperinsulinemia. Thereby, vitamins C and E might have regulated glucose homeostasis through the downregulation of GSK 3β and FOXO1 expression in the liver.

Though the present study did not perform an insulin tolerance test (ITT), an indicative parameter to confirm insulin resistance, glyphosate-exposed hyperinsulinemia may be the reason for the development of insulin resistance and type-2 diabetes shown in the present findings [18]. In this regard, our previous study has shown that glyphosate exposure in a dose-dependent fashion (50, 100, and 200mg/kg body weight) did not reduce blood glucose concentration in response to insulin administration (0.75u/kg. body weight) even after 60 minutes. This study clearly indicates that glyphosate induces hyperglycemic-hyper-insulinemic-insulin resistance. In the current study also 100mg dose of glyphosate caused a significant increase in insulin concentration, which was reduced significantly in Vitamin C and E-treated animals. In accordance with the present study, it has been shown that the administration of antioxidant vitamins (C and E) to DEHP-induced type-2 diabetic rats significantly reduced fasting serum insulin and insulin resistance, and facilitated insulin sensitivity in skeletal muscle [19].

In the human body, GSK3 is present in two isoforms: GSK3∝ and GSK3β. The phosphorylation mediated by GSK3 controls various essential biological activities, encompassing glycogen metabolism, cell signaling, and cellular transport. Notably, GSK3β serves to inhibit glycogen synthesis, leading to reduced glycogenesis in the liver and muscles, thus contributing to hyperglycemia [20-22]. FOXO1 belongs to the group of transcription factors, namely the forkhead family, distinguished by its unique forkhead domain. This transcription factor significantly controls blood glucose levels. It achieves this by overseeing the gluconeogenic and glycogenolytic pathways, which insulin signaling influences [23]. Additionally, FOXO1 contributes to determining the development of adipocytes, directing them toward the process of adipogenesis. The regulation of FOXO1 primarily hinges on phosphorylation across multiple residues [24]. mRNA levels of GSK 3β and FOXO1 were increased in the glyphosate-induced group. This shows us that the glyphosate toxicity will induce changes in the endocrine system.

Vitamin C and E are known for their antioxidant properties [25]. Antioxidants protect against the deleterious effects of oxidative stress and help in the treatment of diseases [26,27]. The present study clearly shows that vitamins C and E have the potential to control GSK 3β and FOXO1 gene-mediated diabetes mellitus in glyphosate-exposed animals. Overall, our findings suggest that diabetic complications that are generated by glyphosate exposure were reduced in treatment with antioxidant vitamins (C and E), which have the capability to reduce the expression of genes involved in diabetic metabolic signaling pathways. Hence, our current study provides in vivo evidence that glyphosate-mediated development of type-2 diabetes via the upregulation of GSK 3β and FOXO1 gene expression in the liver was controlled by the type-2 diabetic rats treated with antioxidant vitamins C and E. Further in vitro cell culture studies employing human cell lines are necessary for providing more evidence for understanding the molecular mechanism underlying glyphosate exposure prior to clinical trials.

## Conclusions

The findings of the present in vivostudy clearly demonstrate that antioxidant vitamins (C and E), reduce hyperglycemia and hyperinsulinemia by upregulating GSK 3β and FOXO1 proteins involved in glucose metabolism and insulin signaling caused by glyphosate exposure. Hence, vitamins C and E may be considered therapeutic antioxidants to combat type-2 diabetes. Further studies on the effects of vitamins C and E on human cell line model in vitro cell lines are warranted to explore molecular mechanisms of action toward the development of clinical trials. 
